# Self-assembly formation of Bi-functional Co_3_O_4_/MnO_2_-CNTs hybrid catalysts for achieving both high energy/power density and cyclic ability of rechargeable zinc-air battery

**DOI:** 10.1038/srep33590

**Published:** 2016-09-20

**Authors:** Nengneng Xu, Yuyu Liu, Xia Zhang, Xuemei Li, Aijun Li, Jinli Qiao, Jiujun Zhang

**Affiliations:** 1 Institute of Sustainable Energy, Shanghai University, 20 Chengzhong Road, Shanghai 201800, China; 2 College of Environmental Science and Engineering, Donghua University, 2999 Ren’min North Road, Shanghai 201620, China; 3 Energy, Mining & Environment, National Research Council of Canada, Vancouver, BC, Canada

## Abstract

α-MnO_2_ nanotubes-supported Co_3_O_4_ (Co_3_O_4_/MnO_2_) and its carbon nanotubes (CNTs)-hybrids (Co_3_O_4_/MnO_2_-CNTs) have been successfully developed through a facile two-pot precipitation reaction and hydrothermal process, which exhibit the superior bi-functional catalytic activity for both ORR and OER. The high performance is believed to be induced by the hybrid effect among MnO_2_ nanotubes, hollow Co_3_O_4_ and CNTs, which can produce a synergetic enhancement. When integrated into the practical primary and electrochemically rechargeable Zn-air batteries, such a hybrid catalyst can give a discharge peak power density as high as 450 mW cm^−2^. At 1.0 V of cell voltage, a current density of 324 mA cm^−2^ is achieved. This performance is superior to all reported non-precious metal catalysts in literature for zinc-air batteries and significantly outperforms the state-of-the-art platinum-based catalyst. Particularly, the rechargeable Zn-air battery can be fabricated into all-solid-state one through a simple solid-state approach, which exhibits an excellent peak power density of 62 mW cm^−2^, and the charge and discharge potentials remain virtually unchanged during the overall cycles, which is comparable to the one with liquid electrolyte.

The increasing global energy usage, its associated fast fossil fuel depletion, and the resulted environmental concern have stimulated intensive research and development of clean and sustainable energy storage and conversion systems^1^,^2^,^3^. Among different energy storage and conversion options, electrochemical energy devices including batteries, fuel cells, and supercapacitors have been recognized as the most feasible and efficient ones. In the most recent years, the rechargeable metal–air batteries, which have a low fabrication cost, environmentally benign, and high safety, have received much interest because of their extremely higher energy density than any commercially available aqueous batteries and lithium-ion batteries^4^,^5^, making them one of the most promising candidates in energy storage and conversion, particularly for extending driving range of the next generation electric vehicles^6^,^7^,^8^. For example, Zinc-air batteries could achieve a specific energy density in excess of 400 Wh kg^−1^(650 Wh L^−1^) in a coin-cell configuration^9^,^10^,^11^. However, the electrically rechargeable Zn-air batteries face the challenge of sluggish kinetics of both oxygen reduction reaction (ORR) and O_2_ evolution reaction (OER) at the positive (cathode) electrode, which can lead to low round trip efficiency. In attempts to improve both the ORR and OER processes, carbon-supported precious metal-based bifunctional electrocatalysts such as Pt, Pd, Ag, Au, Ir and their alloys have been used to increase the rate of the reactions^12^,^13^,^14^,^15^,^16^,^17^. However, both these metals’ high-cost/scarcity and insufficient stability make the technologies impracticable, particularly for a large-scale commercialization^18^. Therefore, developing non-precious metal-based materials such as metal oxides have become one of the important options for bi-functional catalysts for Zn-Air batteries.

Among different bi-functional catalysts explored for metal-air batteries, manganese oxides (MnO_X_) are a kind of the particularly interesting candidates due to their rich oxidation states, chemical compositions and crystal structures. As identified, manganese oxides have high catalytic activity for the decomposition of H_2_O_2_ induced by the simultaneous oxidation and reduction of the surface manganese ions (i.e., Mn^4+^/Mn^3+^ for the mixed manganese based catalyst)^19^. For example, MnO_2_ is the most commonly used ORR electro-catalyst in commercial zinc–air batteries^20^. Besides MnO_X_, Co_3_O_4_, one of the well-known spinel materials, has also been studied for decades as a highly efficient and corrosion-resistant ORR/OER catalyst in alkaline media^21^. To further improve the electrochemical activity of Co_3_O_4_ electro-catalysts, other metal atoms were also incorporated into the spinel structure to form M_X_Co_3−X_O_4_ (M = Ni/Mn/Cu/Li) catalysts in which when M = Ni/Mn, the materials served as the ORR catalysts, and when M = Cu/Mn/Li, the materials as the OER catalysts^22^,^23^,^24^,^25^,^26^,^27^,^28^. Although the incorporation of these metals into Co_3_O_4_ could increase the number of catalytically active sites^6^,^29^, the catalyst’s electrical conductivity was found to be insufficient.

Recent studies reported that the combination between oxides and nano-structural carbons such as graphene and carbon nanotubes (CNTs) to form ORR catalysts could have both improved electro-catalytic activity and stability^28^,^30^,^31^. This could be attributed to their high conductivity, large surface area, and high electrochemical stability^32^,^33^,^34^. To the best of our knowledge, there has been no report on the combination among MnO_2_, Co_3_O_4_ and carbon-based materials to form hybrid catalysts for improving the electro-catalytic performance of the materials in rechargeable metal-air batteries. It is expected that by combining the transition metal oxides with carbon-based material via a facile hydrothermal process would lead to a strong hybrid effect to enhance the catalytic performance. Based on this strategy, we have designed and synthesized the hybrid material via a facial two-pot method where the precursors of the metal-oxide (Mn and Co) and CNTs are justly mixed into a single reaction to produce the final catalysts.

The two-pot synthesis reported in this paper is based on a hydrothermal process, which is simple and cost effective, thus allowing highly practical and scalable preparation of the catalyst material. The synthesized novel hybrid material composed of Co_3_O_4_ supported on MnO_2_ nanotubes (Co_3_O_4_/MnO_2_) and its CNTs hybrid (abbreviated as Co_3_O_4_/MnO_2_-CNTs) have been explored as the bi-functional catalysts, and the tested results show that these catalysts have very high ORR/OER activities and stability. It is believed that the electro-catalytic activities of these catalysts are contributed by the novel phases of the inorganic nano-particles and their intimate to the underlying CNT networks. Using this hybrid electro-catalyst (Co_3_O_4_/MnO_2_-CNTs) for the air cathode, a rechargeable Zn-air battery is constructed for catalyst validation. Compared to baseline catalyst, both remarkable high recharge ability and durability of a practical zinc-air battery are demonstrated by utilizing atmosphere air as the source of oxidant instead of pure purged oxygen, which are even better than the Pt/C catalyst in alkaline solutions. In particular, the significantly reduced ORR and OER over-potentials are achieved, resulting in both high battery performance (a discharge peak power density of 450 mW cm^−2^ and a current density 324 mA cm^−2^ at 1.0 V are achieved) and high durability (long cycle-life), demonstrating that this Co_3_O_4_/MnO_2_-CNTs catalyst is also one of the most promising non-precious catalysts, particularly for the OER.

## Experiment Methods

### Synthesis of MnO_2_ nanotube-supported Co_3_O_4_ and its carbon nanotube hybrid cathode catalyst

A modified facile two-pot hydrothermal method was used to synthesize Co_3_O_4_/MnO_2_-CNTs hybrid cathode catalyst for rechargeable zinc-air batteries. All chemicals used in this work were analytical grade and used without further purification. For a typical synthetic experiment, Solution A was synthesized by dissolving 0.790 g of KMnO_4_ and 2 mL of concentrated HCl (37%) in 50 mL of deionized water with stirring. Then the solution was transferred into a 100 mL Teflon-lined stainless steel autoclave and sealed and hydrothermally treated at 140 °C for 12 hours. The collected powder of MnO_2_ nanotubes was treated by several centrifuge-wash cycles with ethanol and deionized water, and then dried in air at 70 °C for 24 hours. Solution B was prepared by adding 0.25 g of Co(NO_3_)_2_ 4H_2_O in 30 mL of 1.3 mol L^−1^ ammonia solution. Then 0.25 g as-prepared MnO_2_ nanotube powder made from Solution A and 0.125 g CNTs (purity > 95wt.%, length ~15 μm, diameter 30–50 nm, Alpha Nano Technology Co. Ltd., China) were dispersed in Solution B by ultrasonication for 1 hour. This mixture was then transferred into a 100 mL autoclave, which was sealed and maintained at 150 °C for 5 hours. The precipitate was separated by several centrifuge-wash cycles with deionized water, and then dried at 60 °C for 6 hours. The collected powder of Co_3_O_4_/MnO_2_-CNTs hybrid materials were calcined in air at 400 °C for 1 hour to obtain the product. For a comparison, MnO_2_ nanotubes-supported hollow porous Co_3_O_4_ nanomaterials (Co_3_O_4_/MnO_2_) were also prepared under the same procedure expect the CNTs was not added.

### Material characterization

X-ray diffraction (XRD) was used to identity the phase composition of synthesized Co_3_O_4_/MnO_2_-CNTs sample over the 2θ range from 5° to 80° using a Bruker AXS D8 advance diffractometer with nickel filtered Cu K*α* radiation (λ = 1.5406 Å), and the energy-dispersive X-ray (EDX) spectra were taken by a JEOL JSM5600 scanning electron microscope at an accelerating voltage of 20 kV. The microstructural characteristics of Co_3_O_4_/MnO_2_-CNTs hybrid samples were recorded by transmission electron microscopy (TEM), high-resolution transmission electron microscope (HR-TEM, JEOL JEM-2010F) working at 200 kV accelerating voltage, and the lattice structure was identified by selected area electron diffraction (SAED) technique.

### Electrode preparation and electrochemical characterization

A half-cell setup containing a rotating disc electrode (RDE) was used to investigate both the ORR and the OER catalytic activities of the Co_3_O_4_/MnO_2_-CNTs catalyst samples. The working electrode was fabricated by casting Nafion^®^-impregnated catalyst ink onto a glassy carbon disk electrode (5 mm in diameter). In the preparation of catalyst ink, 10 mg of the catalyst was ultrasonically dispersed into 1 mL ethanol and 8 μL 5 wt% Nafion^®^ solution to form a catalyst ink. Then, 5 μL of the catalyst ink was deposited onto the disk and dried at room temperature. The working electrode was allowed to achieve a catalyst loading of 0.1 mg cm^−2^. Electrochemical activity of the samples was studied using a linear sweep voltammetry. In the measurements, the catalyst-coated working electrode was immersed in a half-cell containing 0.1 M KOH aqueous electrolyte, in which a platinum foil and a saturated calomel electrode (SCE) were used as the counter and reference electrodes, respectively. Catalyst activities toward both ORR and OER were evaluated in oxygen-saturated electrolyte solution in a potential range from 1.67 to 0.1 V vs RHE. The rotation rate was controlled at 1600 rpm. A commercial Pt/C catalyst (30 wt% platinum on carbon, Johnson Matthey) and CNTs were used as the baselines and tested using the same procedure as that for Co_3_O_4_/MnO_2_-CNTs catalyst.

### Single cell test

**Figure 1:**
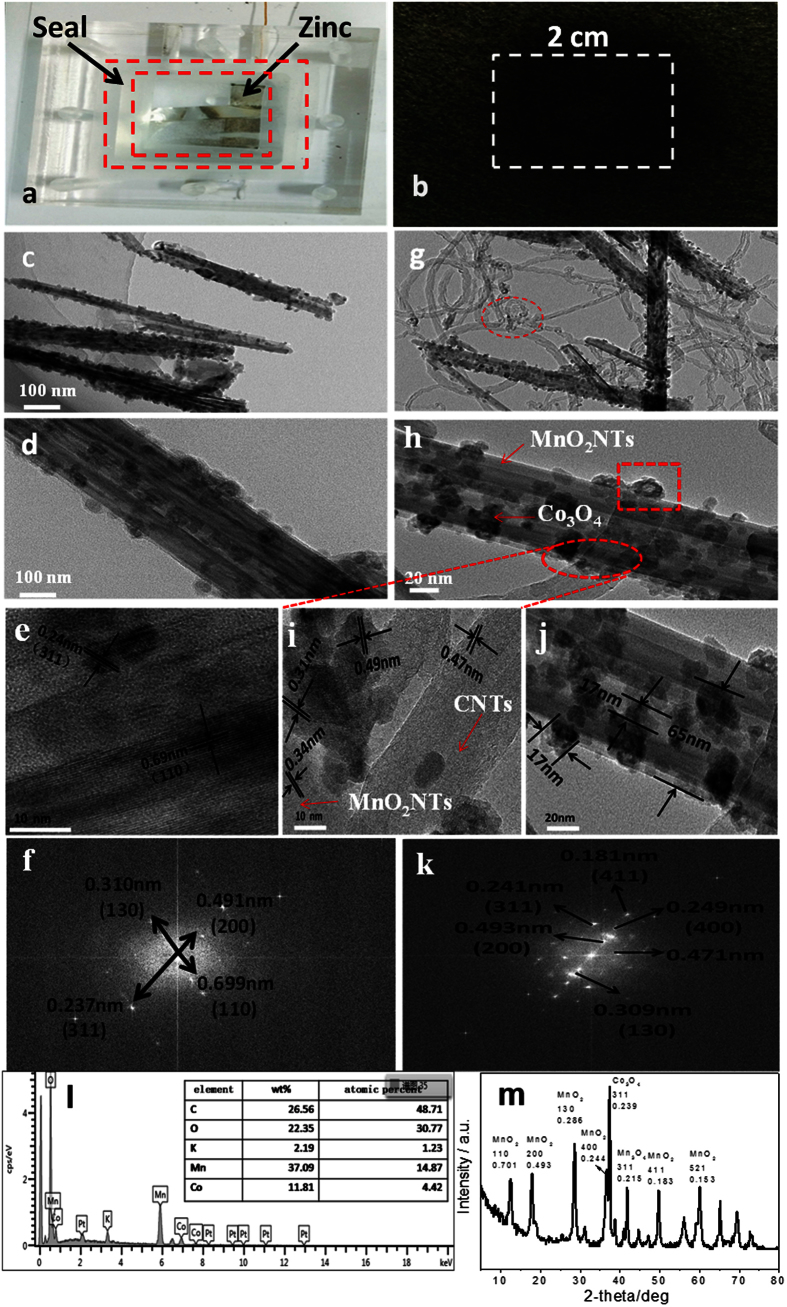
(**a**) Optical image of the home-made rechargeable zinc–air battery; (**b**) Optical image of Co_3_O_4_/MnO_2_-CNTs hybrid catalyst-based air electrode; (**c**) TEM image of Co_3_O_4_/MnO_2_hybrid material; (**d**) TEM image of Co_3_O_4_/MnO_2_ uniformly distributed local graph; (**e**) HRTEM image of the interface of Co_3_O_4_/MnO_2_ nanocrystals; (**f**) FFT pattern of Co_3_O_4_/MnO_2_ in the hybrid; (**g**) TEM image of Co_3_O_4_/MnO_2_-CNTs hybrid catalyst; (**h**) TEM image of Co_3_O_4_/MnO_2_-CNTs uniformly distributed local graph; (**i**) HRTEM image of the interface of Co_3_O_4_/MnO_2_-CNTs nanocrystals; (**j**) HRTEM image of the Co_3_O_4_/MnO_2_-CNTs nanocrystals; (**k**) FFT pattern of Co_3_O_4_/MnO_2_-CNTsin the hybrid; (**l**) EDS spectrum of the Co_3_O_4_/MnO_2_-CNTs hybrid nanomaterials (Insert: composition of the Co_3_O_4_/MnO_2_-CNTs); (**m**) XRD patterns of the Co_3_O_4_/MnO_2_-CNTs hybrid nanomaterials.

A home-made zinc–air battery, as shown in Fig. 1a, was used to validate the practical catalyst activity and stability. The air cathode was prepared by spraying the catalyst (with a loading of 2 mg cm^−2^) onto a gas diffusion layer (GDL) (Toray TGP-H-090) with an exposed active area of 4.0 cm^2^ (Fig. 1b). Briefly, 20 mg of catalyst was dispersed in 5 mL of ethanol by sonication for 30 minutes. 40 *μ*L of 5 wt% Nafion^®^ solution was added followed by 1 hour of additional sonication. The catalyst mixture was then sprayed onto the GDL, and dried in an oven at 60 °C for 1 hour. The catalyst loading was controlled by calculating the weight of the GDL electrode before and after the spray coating. The electrolyte used in the zinc–air battery was 10 mL of 6 M KOH, and a polished zinc plate (purity > 99.99%, thickness: 0.3 or 1.0 mm, Shengshida Metal Mater. Co. Ltd., China) was used as the anode. The discharge polarization and power density plots were obtained using a galvanodynamic method with a current density ranging from 0 to 1000 mA. Battery testing and cycling experiments were performed using the recurrent galvanic pulse method, where one cycle was consisted of a discharging step (5∼10 mA cm^−2^ for 10∼30 minutes, 30∼50 mA cm^−2^ for 2 hours and 100 mA cm^−2^ for 30 minutes) followed by a charging step with the same current and duration time.

For all-solid-state Zinc–Air battery, the battery is fabricated by laminating an Tokuyama A901 anion-exchange membrane (Tokuyama A901 is a major type of commercially available membrane, which exhibits high OH^−^ conductivity of 11.4 mS cm^−1^, ion exchange capacity of 1.7 meq g^−1^, and generally used as a reference for comparing membrane characteristics and fuel cell performances) between an air electrode made of the above Co_3_O_4_/MnO_2_-CNTs catalyst-loaded gas diffusion layer (GDL) (Toray TGP-H-090) and a polished zinc plate anode. Copper (Cu) foil as a substrate is attached to the zinc electrode to ensure a good conductivity. The assembled device was pressed under a pressure of 3 MPa for 1 minute by a sheeting presser to enhance the integrity of the laminated structure.

## Results and Discussion

The morphology information for the catalysts can be seen from the TEM images shown in Fig. 1(c). This figure indicates that MnO_2_ nano-tubes can be successfully synthesized using the facile two-pot method (Figure S1(a)), where MnO_2_ nanotubes consisting of the average diameter and length of 45∼65 nm and 1∼2 μm, respectively. On the surface of MnO_2_ nanotubes, the homogeneous Co_3_O_4_ nano-particles with an average size of 5∼10 nm are densely coated (Fig. 1(d) and Figure S1(b)). The high resolution transmission electron microscopy (HR-TEM) image of the interface between a single MnO_2_ nanotube and Co_3_O_4_ nanocrystal (Fig. 1(e)) reveals fringes in multiple directions with d-spacing of 0.24 nm for cubic Co_3_O_4_ and a lattice spacing of 0.69 nm for α-MnO_2_. The Co_3_O_4_ nanoparticles exhibit an ecumenical crystalline structure as confirmed by the distinct diffraction dots observed in the fast Fourier transformation (FFT) (Fig. 1(f)). Further analyzing the FFT pattern reveals the characteristic lattice spacing of 0.699 nm, 0.491 nm, 0.310 nm and 0.237 nm for the Co_3_O_4_/MnO_2_ sample, indicative of the polycrystalline nature of this material. The typical TEM images as shown in Fig. 1(g,h) confirm the successful synthesis of Co_3_O_4_/MnO_2_-CNTs hybrid material using the simple chemical route in this work. The TEM image shows that the diameter of a α−MnO_2_ tube is about 45∼65 nm, and both the length and the wall thickness are almost the same as that without CNTs junction (Fig. 1(c,d)). This strongly demonstrates that an uniform and dense coating of the Co_3_O_4_ nanoparticles on the surface of MnO_2_ nanotubes can be achieved even in the presence of CNTs. Interestingly, the Co_3_O_4_ nanoparticles seem to be in a highly “hybrid” porous structure with 3∼5 nm of circular hollow centers (boxed in Fig. 1(h) and Figure S1(c)), which may be ascribed to the doping effect during the formation of Co_3_O_4_/MnO_2_-CNTs hybrid. The cobalt oxide nanoparticles are found to mainly stay on the surface of manganese dioxide nanotubes, except for some particles deposited on CNTs’ surface (circled in Fig. 1(g)). Then, 5 nm of cobalt oxide particles could slowly reunite to about 20 nm cobaltosic oxide particles at 400 °C. This unique structure may lead to a high surface area per unit volume, which could create more active sites for the enhanced electro-catalytic oxygen reduction reactions (Fig. 1(h)). The HR-TEM image in Fig. 1(i) reveals that Co_3_O_4_ nanoparticles are not only binding on the manganese dioxide surface, but also supported on carbon nanotube. More importantly, cobalt oxide crystal aggregation could result in three materials linked together. This coupling effect among Co_3_O_4_, MnO_2_ nanotubes and CNTs may allow a better diffusion of reactants through the empty spaces between the neighboring CNTs to induce a high active material utilization^35^. Further from the high resolution images of MnO_2_ nanotubes, Co_3_O_4_ nanocrystals and CNTs, measured by HRTEM (Fig. 1(j)), the lattice spacing of 0.69 nm of a-MnO_2_ can be assigned to the (110) plane of this material^33^ (Joint Committee on Powder Diffraction Standards file no. 41-0141), and the lattice fringe of the (311) plane with a lattice spacing of 0.24 nm to typical Co_3_O_4_ nanoparticles (Joint Committee on Powder Diffraction Standards file no. 76-1802). Both are in a well agreement with those of Co_3_O_4_/MnO_2_ observed in Fig. 1(e). Further analyzing the FFT pattern (Fig. 1(k)) can give more characteristic lattice spacing of 0.181 nm, 0.241 nm and 0.493 nm for the hybrid Co_3_O_4_/MnO_2_-CNTs, which is in a good consistence with the XRD diagram discussed below.

The resulting Co_3_O_4_/MnO_2_-CNTs sample was further characterized by energy-dispersive X-ray spectroscopy (EDS) (Fig. 1(l)). Compositional analysis of the Co_3_O_4_/MnO_2_-CNTs gives that the CNTs, O, Mn and Co contents are 26.56, 22.35, 37.09 and 11.81 wt%, respectively, and that the C, O, Mn and Co atomic percentages (at%) are 48.7, 30.77, 14.87 and 4.42, respectively (Fig. 1(l), Inset). Based on these data, it can be calculated that the formulas of the metal oxides match well with the Co_3_O_4_ and MnO_2_. From the X-ray diffraction (XRD) patterns of Co_3_O_4_/MnO_2_-CNTs as shown in Fig. 1(m), one can see that all major diffraction peaks match well with the standard peaks of tetragonal α-MnO_2_ for the nanotube sample. For Co_3_O_4_/MnO_2_-CNTs hybrid material, the diffraction peaks at 12.6°, 17.9°, 28.7° and 36.7^o^ can be indexed to (110), (200), (130) and (400) planes of MnO_2_, and the diffraction peak at 36.9^o^ can be indexed to (311) plane of Co_3_O_4_, respectively. There are also some peaks that are not labelled, which are possibly related to the various intermediates that are generated in the junction (Fig. 1(m)). In addition, the d-spacing of each crystal orientation observed in the XRD pattern is also calculated based on the diffraction angle using Bragg’s law, the result obtained matches closely with those calculated with the SAED pattern from the TEM characterization (as listed Table 1), confirming the successful synthesis of Co_3_O_4_/MnO_2_-CNTs hybrid catalyst.

**Figure 2:**
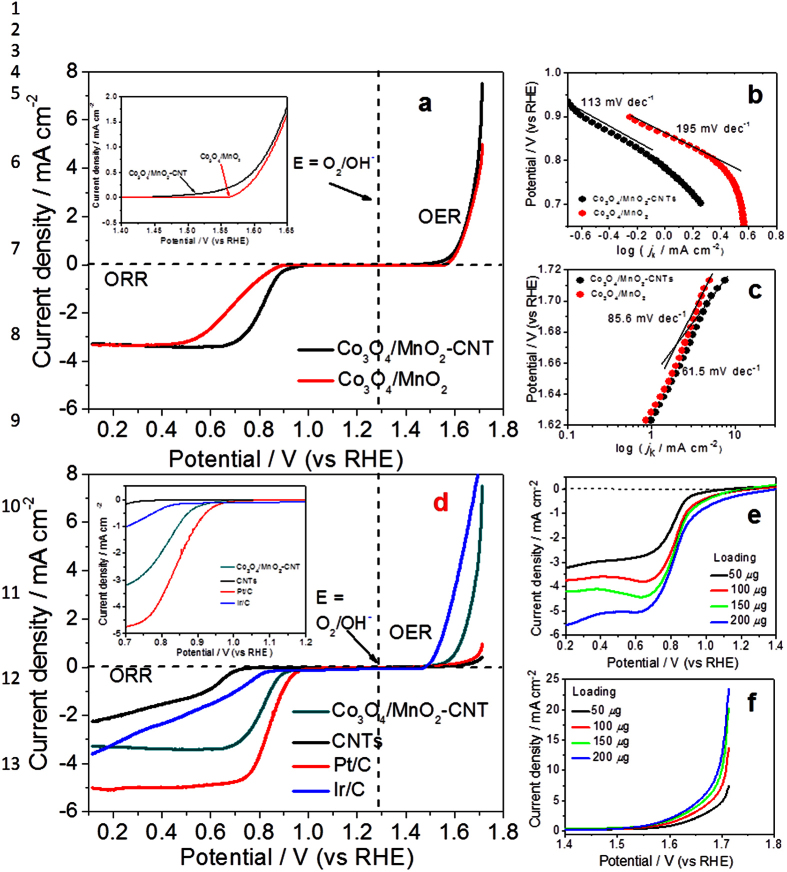
(**a**) ORR and OER polarization curves of Co_3_O_4_/MnO_2_and Co_3_O_4_/MnO_2_-CNTs (Insert: enlarged OER); (**b**) Tafel plots of ORR currents; (**c**) Tafel plots of OER currents; (**d**) ORR and OER polarization curves of Co_3_O_4_/MnO_2_-CNTs, 20%Pt/C, Ir/C and CNTs (Insert: enlarged ORR); (**e**) ORR polarization curves catalyzed by different loadings of Co_3_O_4_/MnO_2_-CNTs catalyst; (**f**) OER polarization curves catalyzed by different loadings of Co_3_O_4_/MnO_2_-CNTs catalyst.

The catalytic activity of the as-prepared Co_3_O_4_/MnO_2_-CNTs catalyst toward both ORR and OER were tested by linear sweep voltammetry (LSV) using a catalyst-coated rotating disk electrode. Figure 2(a) shows the LSV curves of as-prepared Co_3_O_4_/MnO_2_-CNTs hybrid in comparison with the Co_3_O_4_/MnO_2_ one. It can be seen that on the cathodic branch, Co_3_O_4_/MnO_2_-CNTs exhibits a much higher ORR performance than Co_3_O_4_/MnO_2_. where the Co_3_O_4_/MnO_2_-CNTs gives an onset potential ∼95 mV and a half-wave potential ∼94 mV more positive than that of Co_3_O_4_/MnO_2_, respectively, indicating the importance of CNT injunction. The difference in half-wave potentials may be caused by a thin-film quality difference of the two samples. Further analyzing Tafel slopes (Fig. 2b) reveals that the Tafel slopes at low over potentials for Co_3_O_4_/MnO_2_-CNTs and Co_3_O_4_/MnO_2_ are 113 and 195 mV per decade, respectively, suggesting the former has a much high catalytic activity than the latter. The large difference in the Tafel slopes may indicate the difference in their rate limiting steps On the basis of the onset potentials, half-wave potentials and Tafel slopes, one can conclude that Co_3_O_4_/MnO_2_-CNTs hybrid catalyst is much more active than Co_3_O_4_/MnO_2_ for the ORR. The increased ORR activity could be attributed to the beneficial effect of CNTs due to their excellent conductivity, large surface area and networking effect as discussed above (Fig. 1(h,i)). As in metal-air batteries, apart from the ORR activity of the bi-functional catalyst, the excellent OER activity is particularly critical. As shown in Fig. 2(a), Co_3_O_4_/MnO_2_-CNTs hybrid catalyst delivers an OER current density of 7.8 mA cm^−2^ at 1.7 V, which is 1.6 times higher than that of Co_3_O_4_/MnO_2_. Similar characteristics of onset potential can also be obtained, where Co_3_O_4_/MnO_2_-CNTs shows a ∼90 mV more positive than that of Co_3_O_4_/MnO_2_ for OER (Fig. 2(a), Inset). The measured Tafel slope for Co_3_O_4_/MnO_2_ is 85.6 mV per decade, which was greatly reduced to 61.5 mV per decade if Co_3_O_4_/MnO_2_-CNTs catalyst is used, indicative of superior catalytic activity of Co_3_O_4_/MnO_2_-CNTs hybrid to Co_3_O_4_/MnO_2_ even for OER (Fig. 2(c)). For further comparison, Fig. 2(d) shows the linear sweep potential measurements with Co_3_O_4_/MnO_2_-CNTs, CNTs, Ir/C and the commercial Pt/C nanoparticles tested at the same conditions. It is encouraging to note that the Co_3_O_4_/MnO_2_-CNTs hybrid catalyst exhibits a high ORR onset potential of 0.958 V, which is only ∼36 mV more negative than that of Pt/C catalyst (with an onset potential of 0.994 V), and the ORR current is also much larger than those of both CNTs and Ir/C (with an onset potential of only 0.720 V and 0.838 V, respectively). At 0.2 V, Co_3_O_4_/MnO_2_-CNTs can give a catalytic ORR current density of 3.8 mA cm^−2^ along with a defined diffusion-limiting current plateau, which is slightly less than that of Pt/C catalyst (with a defined diffusion-limiting current plateau of 5.1 mA cm^−2^). The excellent OER activity of the Co_3_O_4_/MnO_2_-CNTs hybrid catalyst is further confirmed by the measured onset potential of 1.454 V, which is ∼50 mV more negative than Pt/C catalyst, ∼200 mV more negative than CNTs and only ~26 mV more positive than Ir/C. The OER current densities catalyzed by Co_3_O_4_/MnO_2_-CNTs, Ir/C, CNTs and Pt/C at 1.7 V are 7.9 mA cm^−2^, 8.0 mA cm^−2^, 0.4 mA cm^−2^ and 0.9 mA cm^−2^, indicating that this Co_3_O_4_/MnO_2_-CNTs can give 20 and 8.5 times higher ORR activity than CNTs and Pt/C. It should be mentioned that for OER, the state-of-the-art catalysts are carbon-supported Ir. From Fig. 2(d), it can be seen that the Co_3_O_4_/MnO_2_-CNTs hybrid catalyst is close to Ir/C. These results suggest that Co_3_O_4_/MnO_2_-CNTs is not only an excellent ORR catalyst but also an outstanding OER catalyst. The high activities of the Co_3_O_4_/MnO_2_-CNTs bi-functional hybrid catalyst for both and ORR and OER suggest that the injunction of CNT may change the OER mechanism due to the unique architectures induced from the synergistic effect and the interface effect among the MnO_2_ nanotubes, Co_3_O_4_ nanoparticles and CNTs. One should emphasize that the synthesis sequence is very important for Co_3_O_4_/MnO_2_-CNTs hybrid formation. Unlike the two-pot method-assisted growth of MnO_2_ nanotubes coupling with CNTs, only the aggregated Co_3_O_4_ nanoparticles could be observed if the CNTs were firstly inducted into KMnO_4_ precursor solution. No formation of MnO_2_ nanotubes could be realized and the redundant CNTs were even “dissolved” into these particles completely (Figure S2, Supporting Information). In a further set of experiments, Fig. 2(e,f) shows the LSV curves at different loadings of as-prepared Co_3_O_4_/MnO_2_-CNTs catalyst. Obviously, catalyst loading has a strong effect on its performance, that is, the higher the catalyst loading, the higher the catalytic current. Increasing the Co_3_O_4_/MnO_2_-CNTs loading to 200 μg cm^−2^ could contribute the onset potentials of ∼100 mV and ∼50 mV more positive than that of lowest loading of 50 μg cm^−2^ for ORR and OER, respectively. Furthermore, with increasing catalyst loading, the diffusion current is also increased.

**Figure 3:**
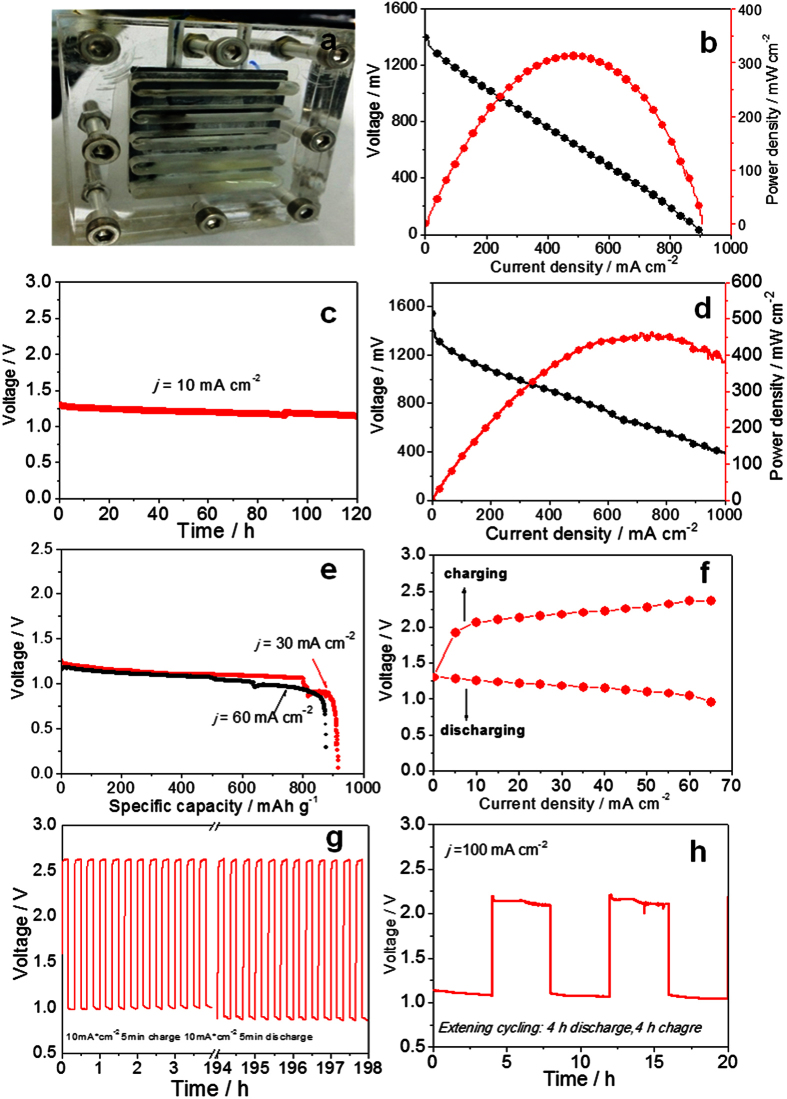
(**a**) Optical image of the already assembled zinc air battery; (**b**) Polarization curve and corresponding power density plot of the Zn-air battery using Co_3_O_4_/MnO_2_-CNTs as the cathode catalyst, with Znic plate with a thickness of 0.3 mm as the anode; (**c**) Long-time discharge curve; (**d**) Polarization curve and corresponding power density plot of the Zn-air battery using Znic plate with a thickness of 1 mm; (**e**) Typical discharge curves of primary Zn-air batteries with Co_3_O_4_/MnO_2_-CNTs as the cathode catalyst under continuous discharge until complete consumption of Zn. Specific capacity was normalized to the mass of consumed Zn. (**f**) Charge and discharge polarization (V-i) curves of the bi-electrode Zn-air battery; (**g**) Cycling data at 10 mA cm^−2^ in cycle periods of 10 minutes per cycle; (**h**) Cycling data at 100 mA cm^−2^ in long cycle periods in 8 hours per cycle.

To validate the catalyst, Co_3_O_4_/MnO_2_-CNTs hybrid catalyst was used as the ORR catalyst loaded on the carbon fibre paper for a cathode of Zn-air battery (Zn foil with 0.3 mm thickness as anode and 6M KOH as the electrolyte) (Fig. 3(a)). It was observed that the assembled battery had an open circuit voltage of 1.40 V. At a cell voltage of 1.0  V, it gave a high current density of 224 mA cm^−2^. The peak power density could be as high as 313 mW cm^−2^ at 0.66 V (Fig. 3(b)), which is significantly superior to those most recently reported Zn-air primary batteries (Table S1, Supporting Information). This Co_3_O_4_/MnO_2_-CNTs cathode catalyst associated primary Zn-air battery was also stable in terms of the performance. When the cell was galvanostatically discharged at a current density of 10 mA cm^−2^ for 120 hours, no obvious voltage drop was observed owing to the stability of Co_3_O_4_/MnO_2_-CNTs for ORR (Fig. 3(c)). It is worthwhile to mention that a higher practical energy density can be easily achieved by simply replenishing the metal anode or electrolyte^21^. To study the durability of air cathode without the failure contribution from battery anode, we tested the cell performance using a zinc plate with a thickness of 1 mm instead of 0.3 mm. Surprisingly, the cell gave a high current density of 324 mA cm^−2^ at a cell voltage of 1.0 V, and a very high peak power density in excess of 450 mW cm^−2^ at 0.7 V (Fig. 3(d)), suggesting that simply replenishing the metal anode or electrolyte could regenerate the battery for subsequent runs at the same performance level with the used Co_3_O_4_/MnO_2_-CNTs cathode. On the contrary, the current density of 100 mA cm^−2^ was only obtained by 20% Pt/C, with a very low peak power density of 140 mW cm^−2^ at the same measuring conditions (Figure S3, Supporting Information). Recently, Zn-air fuel cells or Zn-air flow batteries have been proposed and demonstrated to power electric vehicles with high power, long driving distance and commercial viability^8^,^22^,^36^. They could be quickly refueled with fresh metallic Zn powders (mechanical charging), and the produced zincate species in the electrolyte could be collected and recovered in off-site regeneration facilities. For a battery at 30 mA cm^−2^, the specific capacity normalized to the mass of consumed Znv was ~907 mAh g^−1^, corresponding to a high energy density ~1000 Wh kg^−1^ (Fig. 3(e)). Even for the battery at 60 mA cm^−2^, the specific capacity normalized to the mass of consumed Zn was still ~880 mAh g^−1^, corresponding to a high energy density > 900 Wh kg^−1^ (Fig. 3(e)). Our Co_3_O_4_/MnO_2_-CNTs ORR catalyst should be ideally suited for such a refueling primary Zn-air batteries owing to the exceptional high ORR activity and durability.

Using the cathode catalyst developed in this work, an electrochemically rechargeable Zn-air battery was also constructed and tested. The Co_3_O_4_/MnO_2_-CNTs ink was loaded onto a single cathode for a Zn-air battery for charge and discharge cycling experiments. The electrolyte used was 6 M KOH. Figure 3(f) shows the charge and discharge polarization curves of a rechargeable Zn-air battery. Under different charge and discharge current density, the charge and discharge voltages of zinc air battery have shown good performance. As shown in Fig. 3(f,g), such a battery exhibits a stable cycling stability when charged and discharged galvostatically at controlled current densities (10∼100 mA cm^−2^) vand cycling pattern (10 minute∼8 hours per charge or discharge period). It can be observed that the battery has a much better performance when the charging and discharging currents are small and the cycle time is short (for example, 10 mA cm^−2^, 10 minutes per cycle, in Fig. 3(g)). Even using the extended cycling test (4 hours of discharge followed by 4 hours of charge at 100 mA cm^−2^), the battery can still show both long term durability and narrow charge–discharge voltage gap (~1.0 V) (Fig. 3(h)). It should be noted that the battery oxidant feeding was by an un-enforced atmosphere air instead of pure oxygen or enforced air flow. The results described above are significantly improved over previous reports on Zn-air primary batteries, where oxygen (99.6%) was continuously fed to the cathode during the measurements using 0.2M zinc acetate as electrolyte or the zinc plate being replaced for every certain cycles (Table S2, Supporting Information)^21^,^36^.

**Figure 4:**
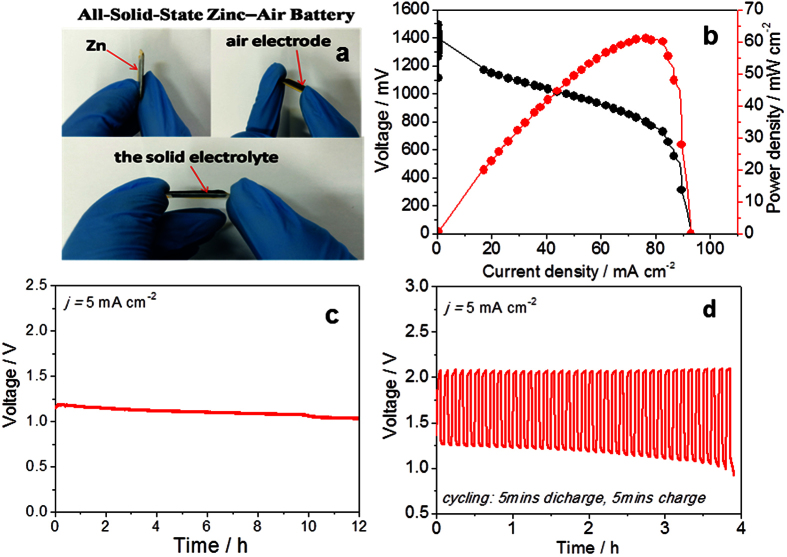
(**a**) A home-made all-solid-state Zinc–Air Battery; (**b**) Polarization curve and corresponding power density plot of the all-solid-state Zn-air battery using Co_3_O_4_/MnO_2_-CNTs as the cathode catalyst, with Znic plate with a thickness of 0.1 mm as the anode; (**c**) Long-time discharge curve; (**d**) Cycling data at 5 mA cm^−2^ in cycle periods of 10 minutes per cycle.

As is known, zinc–air batteries mostly operate in alkaline media, such as 6 M KOH, for the sake of higher activity of both the zinc electrode and air electrode. However, the side-reaction products of K_2_CO_3_ or KHCO_3_, induced by the CO_2_ vin air, can result in the carbon precipitation problem for zinc–air batteries. Moreover, for open systems as zinc–air batteries, water volatilization from the liquid electrolytes is an important cause of performance attenuation. Other issues such as electrolyte leakage and low safety are still the challenges for liquid electrolyte-based metal-air batteries. It has been proposed and evaluated that solid electrolyte can help minimize above challenges^37^. They are also able to suppress the self-corrosion of zinc and eliminate its carbonation. Moreover, using both thin film electrode and polymer electrolyte design can facilitate the physical flexibility of zinc–air batteries, providing remarkable advantages over currently available battery options, and may result in a complete redesign of modern electronics particularly for emerging porvtable and flexible applications. Figure 4(a) shows a fabrication process of the all-solid-state zinc air battery assembly, where the catalyst is loaded on the carbon fibre paper for a cathode, Zn foil with 0.03 mm thickness as the anode, and the Tokuyama membrane as solid electrolyte. The assembled battery shows an open circuit voltage of 1.40 V. Additionally, at a cell voltage of 1.0 V, the cell exhibits a high current density of 100 mA cm^−2^, and the peak power density of 62 mW cm^−2^ (Fig. 4(b)). When the battery is galvanostatically discharged at a current density of 5 mA cm^−2^ for 12 hours, no obvious voltage drop can be observed owing to the stability of Co_3_O_4_/MnO_2_-CNTs for ORR (Fig. 4(c)). Surprisingly, further for the charge and discharge cycling experiments, the discharge (2.125 V) and charge potentials (1.25 V) under the all-solid-state condition remain virtually unchanged during the overall cycles, which is similar to the one with liquid electrolyte. From Fig. 4(d), it can be seen that the battery shows a very good stability in terms of charging. Such a battery exhibits a stable cycling stability when charged and discharged galvostatically at controlled current densities (5 mA cm^−2^) and cycling pattern (5 minutes per charge or discharge period). Compared with thev potential of the charging, only minuscule changes for discharge potentials are observed at last several cycles. The observed potential changes could be due to the delamination of cell components. At this region, the Zn foil was found to be gradually thicken, and more soluble zinc salts were accumulated inside the electrolyte and the Zinc foil. In spite of this, the fabricated all-solid-state cell exhibits stable cycle performance. After 4 hours (24 cycles) operating, the cell voltage is still maintained above 1 V, demonstrating a linear charge–discharge voltage profile. The superior cycling stability and recharge ability of this all-solid-state cell can be attributed to its highly flexible components along with structural integrity between the electrodes and the membrane, and also the improved catalytic activities during the oxygen reactions due to a very strong synergy between Co_3_O_4_/MnO_2_ and NCNT species. A high volumetric energy density (based on the zinc foil volume) corresponding to a gravimetric energy density (based on the zinc foil mass) can be achieved to be 2891 Wh L^−1^ and 597 Wh kg^−1^, respectively. These results are almost 4 times higher than the reported high-energy-density all-solid-state lithium-ion batteries (152 mAh g^−1^)^38^ and Zn–MnO_2_ batteries (308 mAh g^−1^)^39^, demonstratinvg the advancement of such a Co_3_O_4_/MnO_2_-CNTs catalyst-based all-state Zinc-air battery over other devices reported.

## Conclusions

In summary, a MnO_2_ nanotubes-supported Co_3_O_4_ (Co_3_O_4_/MnO_2_) material and its composite with CNTs (Co_3_O_4_/MnO_2_-CNTs) are successfully synthesized through a facile two-pot precipitation reaction and hydrothermal process. This Co_3_O_4_/MnO_2_-CNTs hybrid nanocatalyst is used as a highly active bi-functional catalyst for the oxygen reduction and oxygen evolution reactions in both pvrimary and secondary Zn-air batteries. Experiments show that this bifunctional catalyst has both higher catalytic ORR and OER activities and stability than other baseline materials such as α-MnO_2_ nanotubes, CNTs and even commercially available Pt/C catalysts. The high performance of this novel catalyst is believed to be induced by the hybrid effect among MnO_2_ nanotubes, Co_3_O_4_ and CNTs, which can produce a synergy for enhancing its both catalytic ORR and OER activities and stability. To validate this catalyst material, both primary and electrochemically rechargeable Zn-air batteries are employed, in which Co_3_O_4_/MnO_2_-CNTs is used as the cathode (or positive electrode) catalyst. Particularly, the rechargeable battery shows the high performance with an excellent cycling stability. The maximum power density achieved can be as high as 450 mW cm^−2^. In addition, benefiting from the use of highly flexible electrodes and polymer electrolyte membrane, the rechargeable Zn-air battery can also be fabricated into all-solid-state one through a simple solid-state approach, which exhibits both excellent peak power density and cyclic stability. All of these battery tests have confirmed that this Co_3_O_4_/MnO_2_-CNTs bifunctional catalyst developed in this work has a significant advantage ovevr existing commercial bifunctional catalysts in practical Zn-air batteries.

## Additional Information

**How to cite this article**: Xu, N. *et al*. Self-assembly formation of Bi-functional Co_3_O_4_/MnO_2_-CNTs hybrid catalysts for achieving both high energy/power density and cyclic ability of rechargeable zinc-air battery. *Sci. Rep*. **6**, 33590; doi: 10.1038/srep33590 (2016).

## Supplementary Material

Supplementary Information

## Figures and Tables

**Table 1:** Calculated values of d-spacing for each crystal orientation observed in the SAED pattern obtained from TEM and diffraction angles obtained from XRD of Co_3_O_4_/MnO_2_-CNTs.

Catalysts	Crystal orientation	TEM		XRD	
		d-Spacing (nm)		2 Theta (degree)	d-Spacing (nm)
MnO_2_	110	0.699	12.599		0.701
	200	0.491	17.959		0.493
	130	0.309	28.680		0.311
	400	0.249	36.799		0.244
	411	0.181	49.773		0.183
Co_3_O_4_	311	0.239	36.991		0.239
