# Supplementary material for: Self-assembly formation of Bi-functional Co3O4/MnO2-CNTs hybrid catalysts for achieving both high energy/power density and cyclic ability of rechargeable zinc-air battery
Source: Sci Rep. 2016 Sep 20;6:33590. doi: 10.1038/srep33590 (PMC5028838; doi:10.1038/srep33590)
Supplement: Supplementary Information [file srep33590-s1.pdf]

Supplementary Information for

Self-assembly formation of Bi-functional Co_3O_4/MnO_2-CNTs hybrid catalysts for achieving both high energy/power density and cyclic ability of rechargeable zinc-air battery

Nengneng Xu[a,b], Yuyu Liu[a], Xia Zhang[b], Xuemei Li[b], Aijun Li[a], Jinli Qiao[a,b], Jiujun Zhang[a,c]

[a]*Institute for Sustainable Energy, Shanghai University,149 Yanchang Road, Shanghai 200072,* China

[b]*College of Environmental Science and Engineering, Donghua Universtiy, 2999 Ren'min North Road, Shanghai 201620, China*

[c]*Energy, Mining & Environment, National Research Council of Canada, Vancouver, BC, Canada*

*Corresponding author. Tel: +86-21-67792379. Fax: +86-21-67792159.

E-mail: qiaojl@dhu.edu.cn, Aijun.li@shu.edu.cn, jiujun.zhang@shu.edu.cn

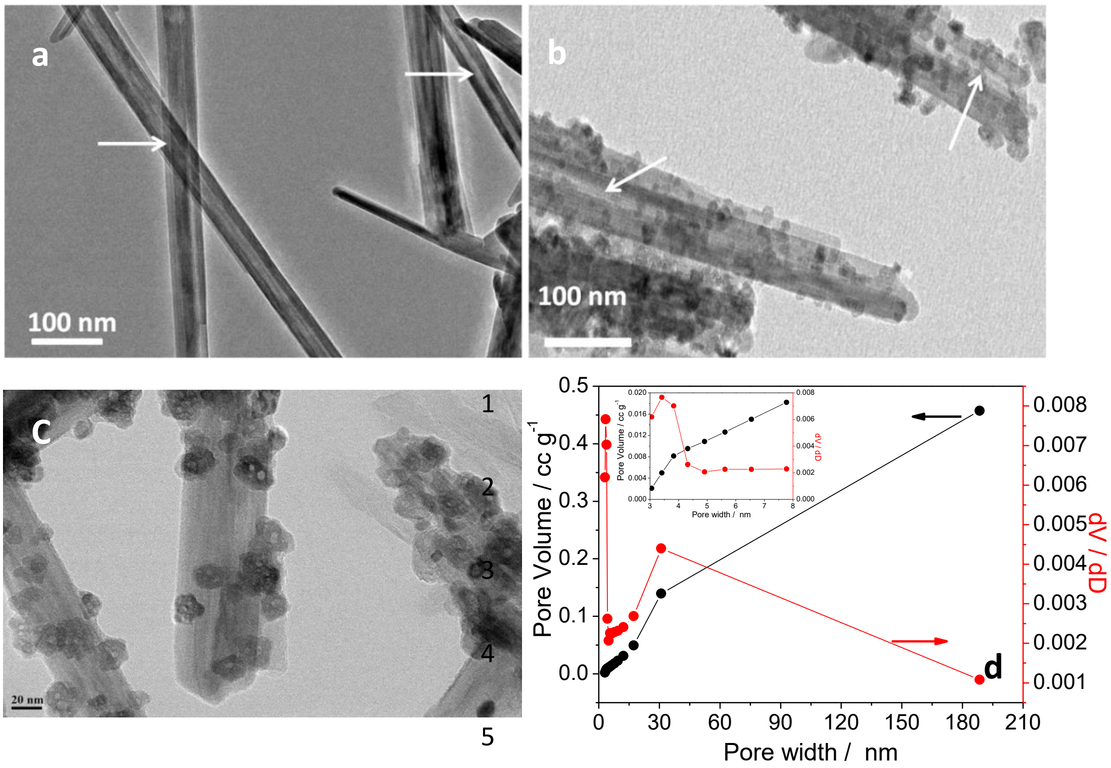

Figure S 1. TEM images of (a) MnO_2 nanotubes, (b) Co_3O_4/MnO_2 nanohybrid materials, (c) Co_3O_4/MnO_2-CNTs nanohybrid materials, (d) pore-size distribution plots derived from N_2-sorption porosimetry

Figure S1 shows the Co_3O_4/MnO_2-CNTs hybrid nanomaterial using the facile two-pot method developed in this work. For obtaining the Co_3O_4/MnO_2-CNTs, the MnO_2 nanotubes (Figure S 1 (a)) were firstly prepared in the first-pot, and then CNTs were added in the second-pot. It can be seen that TEM image exhibits a very clear formation of MnO_2 nanotubes with a diameter of about 45 ~ 65 nm, and on the surface of the MnO_2 nanotubes, the homogeneous Co_3O_4 nanoparticles are densely coted (Figure S 1 (b)). Interestingly, these Co_3O_4 nanoparticles (in a diameter of 20 nm) have a highly hybrid porous structure with many circular hollow centers of diameter 3 ~ 5 nm (Figure S 1(c) and (d)), which may be induced by the doping effect during the formation of the Co_3O_4/MnO_2-CNTs hybrid. This unique structure might lead to a high surface area per unit volume and also more active sites which are highly desirable for electrochemical reactions (Figure 2 in the main text).

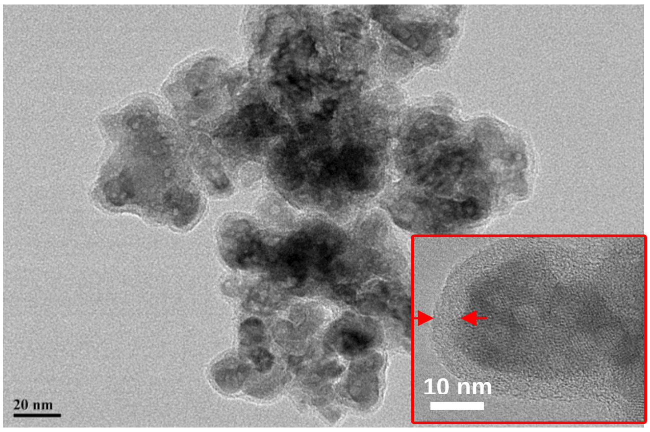

Figure S2. TEM image of Co_3O_4/MnO_2-CNT hybrid materials using different synthesis sequence.

Figure S2 shows the TEM images of the Co_3O_4/MnO_2-CNT hybrid material by changing the synthesis sequence. In a typical synthesis procedure, 0.7902g $KMnO_4$ and 2 mL concentrated HCl were added to 50 mL deionized water to form a precursor solution. Afterwards, 0.50g CNTs were dispersed in the above solution by ultrasonication for 1 hour, then the solution was transferred into a 100 mL Teflon-lined stainless steel autoclave, sealed and hydrothermally treated at 140°C for 12 hours. After the autoclave was cooled down to room temperature naturally, the MnO_2-CNTs hybrid samples were collected and washed with ethanol and deionized water, then was dried in air at 70°C for 24hours. The Co_3O_4 nanoparticles were supported onto the above MnO_2-CNTs hybrid by a hydrothermal method combined with a post-heat treatment. In detail, 0.25 g $Co(NO_3)_2$ $4H_2O$ was dissolved in 15 mL of 1.3 mol L^{-1} ammonia solution, then were dispersed in the above MnO_2-CNTs hybrid solution by ultrasonication for 60 minutes. This mixture was then transferred into a 100 mL autoclave, which was sealed and maintained at 150°C for 5 hours. The resulting product was separated by centrifugation, washed with deionized water, dried at 60°C for 6 hours, and then calcined in air at 400°C for 1 hour.

From the TEM image, only Co_3O_4 nanocrystals can be clearly observed and quite aggregated each other. No MnO_2 nanotubes are formed. Instead, the exposed Co_3O_4 particles seem to be clearly encased in carbon shells with a thickness ~ 5 nm. When CNTs was firstly introduced into the $KMnO_4$ precursor solution, $KMnO_4$ likely to be directly reduced by CNTs to produce the usual MnO_2 nanoparticle instead

of MnO_2 nanotubes. The injuncted CNTs disappeared also, and it seemed to be “dissolved into” the Co_3O_4-MnO_2 nanocrystals completely, which was likely oxidized to CO_2 or formed the thin carbon shell (insert in TEM). At this stage, covalent coupling between Co_3O_4 nanocrystals and MnO_2 were completely destroyed due to the changed structure.

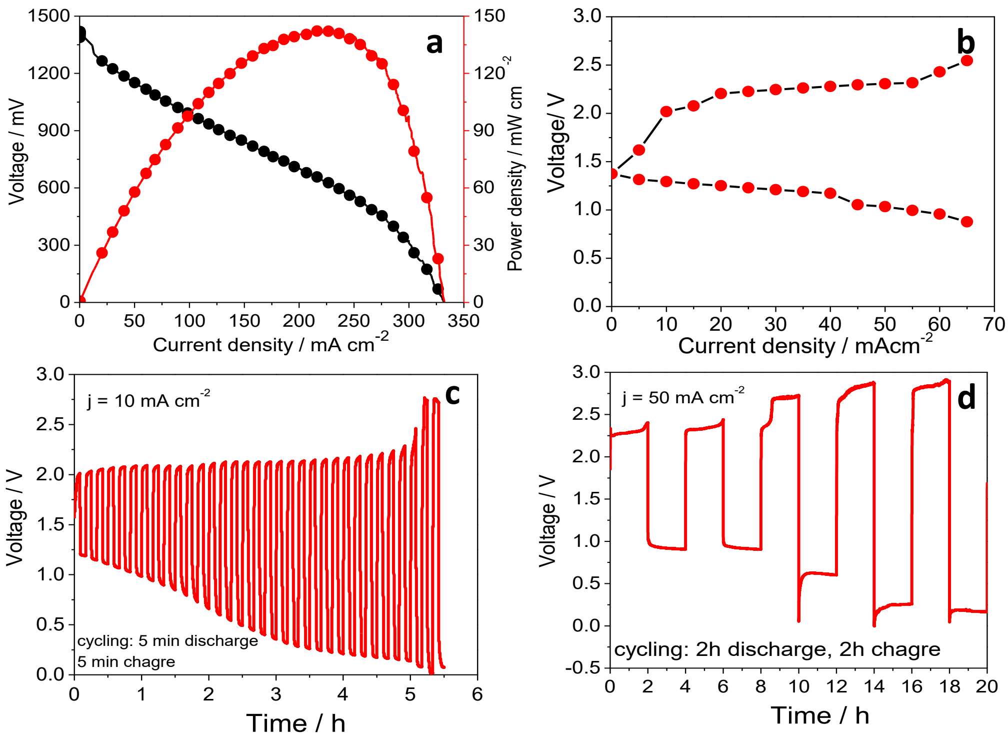

Figure S3. (a) Polarization curve and corresponding power density plot of the Zn-air battery using Pt/C as the cathode catalyst, with Zinc plate with a thickness of 1mm as the anode; (b) Charge and discharge polarization (V-i) curves of the bi-electrode Zn-air battery; (c) Cycling data at 10 mA cm^{-2} in cycle periods of 10 minutes per cycle Long-time discharge curve; (d) Cycling data at 50 mA cm^{-2} in long cycle periods in 4 hours per cycle.

For a comparison, 20% Pt/C was used as the ORR catalyst loaded on the carbon fibre paper for a cathode of Zn-air battery (Zn foil with 1 mm thickness as anode and 6M KOH as the electrolyte). It was observed that the assembled battery had an open circuit voltage of 1.43 V. At a voltage of 1.0 V, it gave a current density of 100 mA cm^{-2}, which is much lower than that of Co_3O_4/MnO_2-CNTs. The peak power density

could be as high as 140 mW cm^{-2} at 0.648V (**Figure S3(a)**). This 20% Pt/C cathode catalyst associated primary Zn-air battery was very normal. Using the cathode catalyst developed in this work, an electrochemically rechargeable Zn-air battery was also constructed and tested. **Figure S3**(a) shows the charge and discharge polarization curves of a rechargeable Zn-air battery. Under different charge and discharge current density, the charge and discharge voltage of zinc air battery has bad performance. As shown in **Figure S3**(c) and (d), such a battery inferior a stable cycling stability when charged and discharged galvostatically at controlled current densities (10 ～ -50 mA cm^{-2}) and cycling pattern (10 minute ～ 4 hours per charge or discharge period). When using the extended currents test with only 2h of discharge followed by 2h of charge at 50 mA cm^{-2}), the battery showed both bad durability and broaden charge–discharge voltage gap (~ 1.7V). Especially the performance of the catalyst at high current of oxygen reduction performance is very poor in **Figure S3**(d). It is worth noting that, after the end of the cycle, charge and discharge voltage difference is larger than 2.6V.

Table S1: Peak power density of primary Zn-air batteries with several key parameters extracted from literatures.

**ORR catalyst used**	**Zn electrode/ electrolyte**	**Current Density @ V = 1.0 V (mA cm^{-2})**	**Peak power density (mW cm^{-2})**	**Reference**
MnO_2/Co_3O_4-CNTs	zinc plate (0.3 mm)/ 6 M KOH	224	313	This work
MnO_2/Co_3O_4-CNTs	zinc plate (1 mm)/ 6 M KOH	250	410	
CoO/N-CNT	Zn foil/6M KOH	197	265	*Nat Commun*. **2013**, 4, 1805
Mn_3O_4/Graphene	Zn powders/-	70	120	*Energy Environ. Sci*. **2011**, 4, 4148
MnOx/Ketjenblack carbon	Zn powders/6M KOH	120	190	*Nano Lett*. **2011**, 11, 5362
MnO_2 nanotubes<br>MnO_2/Co_3O_4 hybrid	zinc plate/6 M KOH<br>zinc plate/6 M KOH	–<br>–	36<br>33	*Nanoscale*, **2013**, 5, 4657<br>*Nanoscale*, **2013**, 5, 4657
MnO_2<br>PbMnOx	zinc sheet /6 M KOH	20<br>30	19<br>38	*Electrochim. Acta*, **2011**, 56, 6205

Table S2: Stability of rechargeable Zn-air batteries with several key parameters extracted from literatures.

Air catalyst used	Cycling conditions and stability	Voltage polarization / V	Reference
Co_3O_4/MnO_2 NTs-CNTs	5-10 mA/cm^2, 10-60 min per cycle period for ~200 cycles	0.8	This work
	30 mA/cm^2, 4h per cycle period for 25 h	0.9	
	100 mA/cm^2, 1h per cycle period for 7 h	1.25	
CoO/N-CNT + NiFe LDH	5-10 mA/cm^2, 200s per cycle period for 60 cycles	0.6	*Nat Commun*. **2013**, 4, 1805
	20 mA/cm^2, 20 h per cycle period for 2 cycles	1.0	
CoO/N-CNT + NiFe LDH Tri-electrode	20 mA/cm^2, 20 h per cycle period for 200 h:6M KOH with 0.2M zinc acetate dissolved to form zincate	0.7	
	50 mA/cm^2, 20 h per cycle period for 40 h: 6M KOH with 0.2M zinc acetate dissolved to form zincate	0.9	
MnO_2 nanotube	15 mA/cm^2, 7 min per cycle period for 60 cycles: polarization increased 0.3~0.4 V at the end	1.4	*Nanoscale*, **2013**, 5, 4657
MnO_2/Co_3O_4 hybrid	15 mA/cm^2, 7 min per cycle period for 60 cycles: polarization increased 0.1~0.2 V at the end	1.0	
MnO_2 nanotube + CNT composite	~8 mA/cm^2, 10 min per cycle periods for 50 cycles: polarization increased ~0.4 V at the end	1.5	*Electrochim. Acta*, **2012**, 69, 295
$LaNiO_3$ supported on N-doped CNTs	~17.6 mA/cm^2, 10 min per cycle period for 75 cycles: polarization increased 0.1~0.2 V at the end	1.2	*Nano Lett*. **2011**, 11, 5362
MnO_2 + stainless steel Tri-electrode	5-15 mA/cm^2, 24-30 h per cycle period for ~120 hrs: negligible voltage change at the end	0.9	*ECS Trans*. **2011**, 28, 25
Co_3O_4 NWs grown on stainless steel electrode	17.6 mA/cm^2, 10 min-6 h per cycle period for ~100 cycles: the zinc plate was replaced every 20 cycles	1.25	*Adv. Energy Mater*. **2014**, 4, 816
